# The Influence of the Strain and Stress Gradient in Determining Strain Fatigue Characteristics for Oscillatory Bending

**DOI:** 10.3390/ma13010173

**Published:** 2020-01-01

**Authors:** Andrzej Kurek, Justyna Koziarska, Tadeusz Łagoda

**Affiliations:** Faculty of Mechanical Engineering, Opole University of Technology, 45-271 Opole, Poland; justyna.koziarska@wp.pl (J.K.);

**Keywords:** fatigue characteristics, bending, tension–compression, stress gradient, strain gradient

## Abstract

In this study, we created a new model to determine strain fatigue characteristics obtained from a bending test. The developed model consists of comparing the stress and strain gradient surface ratio for bending and tensile elements. For model verification, seven different materials were examined based on fatigue tests we conducted, or data available in the literature: 30CrNiMo8, 10HNAP, SM45C, 16Mo3 steel, MO58 brass, and 2017A-T4 and 6082-T6 aluminum alloys. As a result, we confirmed that the proposed method can be used to determine strain fatigue characteristics that agree with the values determined on the basis of a tensile compression test.

## 1. Introduction

Almost every type of industry has been considered in research on the analysis and, ultimately, the prevention of risk, especially in terms of occupational safety. As such, estimating the fatigue limit is one of the most important aspects of strength analysis of structural components. To examine the fatigue limit of various materials, tests must be performed under tension–compression or oscillatory bending, and the test results for the specimens of the occurrence of stresses and strains must be subsequently analyzed. However, the origin of these stresses is not usually considered, although the terms normal or shear stress appears in the analysis of fatigue life. The amplitude of the normal stress *σ_a_* can be derived, for example, from tension–compression, oscillatory bending with restraint, three-point and four-point bending, or rotary bending.

It is important to note that in the case of bending, we always have a linear distribution of the strain gradient, which in the case of elasticity corresponds to the same stress distribution. The situation changes dramatically in the event of plastic deformations. Here, even if the sheet is rolled, the stress distribution is not linear due to various elastic and plastic deformations in the cross-section [1]. Therefore, the problem is much more complicated for bent elements. Plastic deformations appear on the surface, which disappear as they approach the bending plane. The greater these deformations, the more the stress distribution has a gradient that is more and more perpendicular to the surface. In the extreme case of a plastic joint, this distribution approaches the rectangular one in the tension and compression parts, respectively. In addition, in the case of the mesoscopic scale, the plastic deformation gradient significantly affects the corresponding stress gradient [2].

Few studies have paid attention to differences in fatigue resulting from the load [3,4,5,6,7,8,9,10,11,12]. A different fatigue life may correspond, as a result, to the same strain or stress curve. Dorr T. et al. [12] proved that changing the bending plane during tests by π/2 changes the fatigue life as well. None of the cited works have thoroughly analyzed this phenomenon. Although there are theories that consider stress and strain gradients in material fatigue in general, for example, of Gil-Sevillano et al. [13], there are none that use it to better predict fatigue life according to the oscillatory bending tests. Therefore, determining the characteristics using tests at different loads for the same materials is required.

The effect of the stress gradient, and thus the strain gradient, is rarely directly included in fatigue life estimation models. The gradient method is one of the methods of forecasting fatigue limit discussed in the literature [14], where, when using a gradient method, the stress is modified with a relative or absolute stress gradient.

Few attempts have been made to discuss or use the gradient in the literature. In one of the latest publications [15], a strain gradient was used to predict the influence of the microstructure on the initiation of failure, in addition to initial works considering the stress gradient [16,17,18]. The reported results can be used to calculate stress for failures with sharp notches and to assess the fatigue limit of notched components. These works showed that fatigue is significantly influenced by both the load method and geometry, e.g., the notch, where these relationships can be described based on the stress gradient using a dimensionless coefficient, given by:

(1) $$x=\frac{1}{\sigma_{max}}\frac{\partial \sigma_{y}}{\partial_{x}}$$

where *x* is the distance from the bending plane, $\sigma_{max}$ is the maximum stress. The gradient changes with the change in the specimen size. This model was used in previous studies [19].

In this study, seven different materials were used based on selected fatigue tests available in the literature, along with tests conducted by us: 10HNAP, 30CrNiMo8, SM45C, 16Mo3 steel, MO58 brass, and 2017A-T4 and 6082-T6 aluminum alloys.

Fatigue life determined from fatigue tensile–compressing tests indicates lower or comparable values for fatigue life obtained under oscillatory bending [20]. Therefore, let us assume the theory that when using the tensile–compressing model for calculations of fatigue life for oscillatory bending, correct and safe results can be expected [21,22,23].

The results obtained under oscillatory bending conditions represented by the determined secant modulus based on the ratio of the stress gradient and the strain gradient at the critical location, i.e., on the surface, are significantly similar to the results obtained during tension–compression.

## 2. Materials and Methods

### 2.1. Stress and Strain Gradient

Basquin [24] proposed a fatigue graph depicting the dependence of the number of cycles to failure from the stress amplitude in a double logarithmic system $\log\left(\sigma_{a}\right)-\log\left(N_{f}\right)$ and a formula expression for tension–compression in exponential notation can be represented as [6]:

(2) $$\sigma_{a}=\sigma_{f}{\left(2N_{f}\right)}^{b}$$

or in the form of:

(3) $$\log N_{f}=A+m\log\sigma_{a},$$

where *N_f_* is the fatigue life in cycles, $\sigma_{a}$ is the stress amplitude for tension–compression or bending, and *a* and *m* are constants in the regression model.

The basic fatigue characteristic for tension–compression is the Manson–Coffin–Basquin modulus (MCB) [24,25]:

(4) $$\varepsilon_{a,t}=\varepsilon_{a,e}+\varepsilon_{a,p}=\frac{\sigma \prime_{f}}{E}{\left(2N_{f}\right)}^{b}+\varepsilon \prime_{f}{\left(2N_{f}\right)}^{c}$$

where $\varepsilon_{a,t}$ is the amplitude of the total strain expressed by the sum of the amplitudes of the elastic $\varepsilon_{a,e}$ and plastic $\varepsilon_{a,p}$ strain; 2*N_f_* is the number of loading reversals (semi-cycles); E is Young’s modulus; *σ’_f_* and *c* are the coefficient and exponent of the fatigue limit, respectively; and *ε’_f_* and *c* are the coefficient and exponent of the plastic fatigue strain.

**Figure 1:**
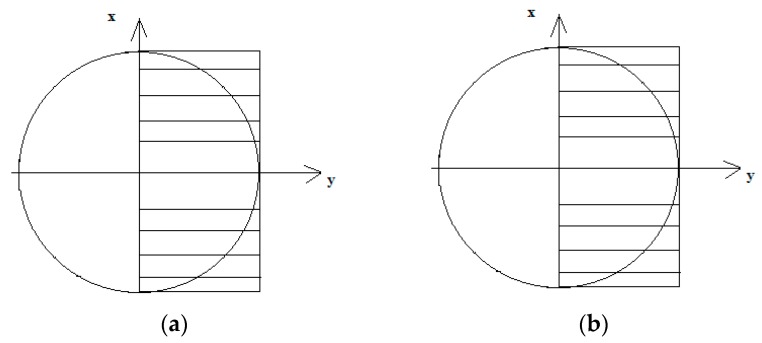
The distribution of (**a**) ε strains and (**b**) *σ* stresses under tension–compression.

For tension–compression, the uniaxial distribution of strains and stresses is as presented in Figure 1.

**Figure 2:**
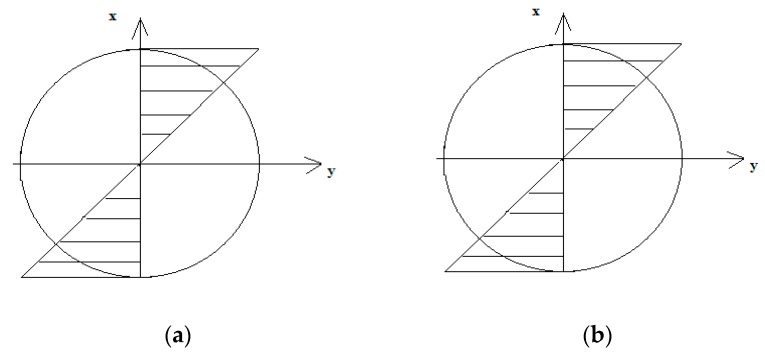
The distribution of (**a**) ε strains and (**b**) *σ* stresses under bending.

In the case of an elastic body model for bending, the distribution of strains and corresponding stresses are linear, as presented in Figure 2.

In the literature, no simple model exists for determining strains and stresses according to the model of the elastoplastic body for specimens without notches when bending. For small strains, the distribution of normal strains in the cross-section for bending was linear [21], and we assumed that this is a geometric condition that must be met first to obtain fatigue values under bending conditions in the elastoplastic model.

(5) $$\varepsilon_{a}\left(x\right)=\varepsilon_{a\;max}\frac{X}{R}$$

where *x* is the distance from the bending plane, and *R* is the maximum height.

The second condition to be met is a physical condition, i.e., a bending moment that must be balanced by the normal stresses:

(6) $$M_{b}=\int_{S}\sigma \left(x,y\right)xdS$$

The Ramberg–Osgood equation combines the stress amplitude with the strain amplitude and is described as [26]:

(7) $$\varepsilon_{a,t}=\varepsilon_{a,e}+\varepsilon_{a,p}=\frac{\sigma_{a}}{E}+{\left(\frac{\sigma_{a}}{K^{\prime }}\right)}^{\frac{1}{n^{\prime }}}$$

where $\varepsilon_{a}$ is the stress amplitude, *K*’ is the coefficient of cyclic strength, and *n*’ is the exponent of cyclic strengthening.

**Figure 3:**
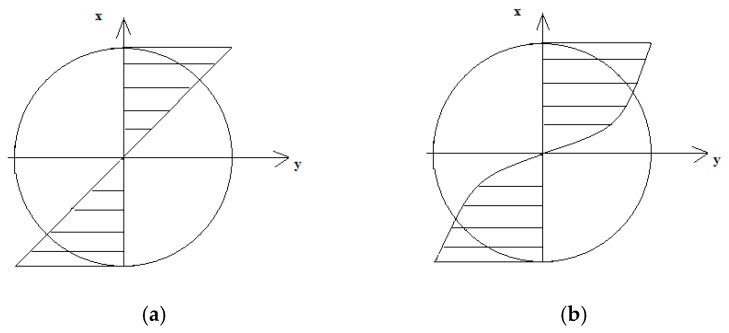
The distribution of (**a**) ε strains and (**b**) *σ* elastoplastic stresses under bending.

In total, the system of equations consisting of conditions in Equations (5)–(7) must be met; on this basis, the elastoplastic strains and appropriate stresses can be determined. The distribution of strains and stresses was shaped as presented in Figure 3. The linear strain gradient corresponded to a non-linear stress gradient.

Using the strain derivative after *x* from Equation (6), we obtained a derivative for strains:

(8) $$\frac{d\varepsilon }{dx}=\frac{\varepsilon_{amax}}{R}$$

In the elastic range, the stress derivative after *x* for bending has the form:

(9) $$\frac{d\sigma }{dx}=\frac{\sigma_{amax}}{R}$$

By using the assumptions in Equations (7) and (8), we obtained the ratio of stress and strain derivatives:

(10) $$\frac{\left(\frac{d\sigma }{dx}\right)}{\left(\frac{d\varepsilon }{dx}\right)}=\frac{\left(\frac{\sigma_{a\;max}}{R}\right)}{\left(\frac{\varepsilon_{a\;max}}{R}\right)}=\frac{\sigma_{a}{}_{max}}{\varepsilon_{a}{}_{max}}=E$$

which in effect, corresponds to Young’s modulus of elasticity.

According to Equations (3) and (4), for a model of the elastoplastic body:

(11) $$\varepsilon \left(x\right)=\varepsilon_{a}{}_{max}\frac{x}{R}=\frac{\sigma_{a}\left(x\right)}{E}+{\left(\frac{\sigma_{a}\left(x\right)}{K^{\prime }}\right)}^{1/n^{\prime }}$$

Counting the derivative on both sides after *x* from Equation (10), we obtained:

(12) $$\frac{\varepsilon_{a}{}_{max}}{R}=\frac{1}{E}\frac{d\sigma_{a}\left(x\right)}{dx}+{\left(\frac{1}{K^{\prime }}\right)}^{1/n^{\prime }}\left(\frac{1}{n^{\prime }}\right)\sigma_{a}{\left(x\right)}^{1/n^{\prime }-1}\left(\frac{d\sigma_{a}\left(x\right)}{d\left(x\right)}\right)$$

and after transformation, we obtained:

(13) $$\frac{\varepsilon_{a}{}_{max}}{R}=\left[\frac{1}{E}+{\left(\frac{1}{K^{\prime }}\right)}^{1/n^{\prime }}\left(\frac{1}{n^{\prime }}\right)\sigma_{a}{\left(x\right)}^{1/n^{\prime }-1}\right]\cdot {\frac{d\sigma_{a}\left(x\right)}{d\left(x\right)}}_{}$$

In other words,

(14) $$\frac{d\sigma \left(x\right)}{d\left(x\right)}=\frac{\frac{\varepsilon_{a}{}_{max}}{R}}{\frac{1}{E}+{\left(\frac{1}{K^{\prime }}\right)}^{1/n^{\prime }}\frac{1}{n^{\prime }}\sigma {\left(x\right)}^{1/n^{\prime }-1}}$$

Assuming that the secant modulus at a given point includes plastic strains, it is defined as:

(15) $$E_{p}=\frac{\frac{d\sigma }{dx}}{\frac{d\epsilon }{dx}}$$

by introducing Equations (8) and (14) into (15), we obtained:

(16) $$E_{p}=\frac{1}{\frac{1}{E}+{\left(\frac{1}{K^{\prime }}\right)}^{\frac{1}{n^{\prime }}}\frac{1}{n^{\prime }}\sigma {\left(x\right)}^{\frac{1}{n^{\prime }}-1}}$$

Eventually, by dividing the sides of Equations (10) and (16), we obtained:

(17) $$\frac{E}{Ep}=1+E{\left(\frac{1}{K^{\prime }}\right)}^{1/n^{\prime }}\frac{1}{n^{\prime }}\sigma_{a}{\left(x\right)}^{1/n^{\prime }-1}$$

where *K*’ is the coefficient of cyclic strength and *n*’ is the exponent of cyclic strengthening.

We proposed the following relationship between the amplitude for oscillatory bending according to the elastoplastic model and the amplitude including the gradient:

(18) $$\sigma_{a,grad}=\sigma_{a,e-p}{\left(\frac{E}{E_{P}}\right)}^{a}$$

where *E* is Young’s modulus, *a* is the exponent of fatigue stress, and $\sigma_{a,e-p}$ is the stress amplitude for bending according to the full elastic model.

In the proposed model, we assume that a in Equation (18) is:

(19) $$a=\frac{n^{\prime }}{5}$$

Eventually, by substituting Equation (17) into Equation (18), we obtained:

(20) $$\sigma_{a,grad}=\sigma_{a,e-p}{\left[1+E{\left(\frac{1}{K^{\prime }}\right)}^{\frac{1}{n^{\prime }}}\frac{1}{n^{\prime }}\sigma_{a}{\left(x\right)}^{\frac{1}{n^{\prime }}-1}\right]}^{\frac{1}{5n^{\prime }}}$$

We obtained the maximum stress on the surface; in other words, for $x=R,\;\sigma_{a}\left(x\right)=\sigma_{a,e-p}$ or:

(21) $$\sigma_{a,grad}=\sigma_{a,e-p}{\left[1+E{\left(\frac{1}{K^{\prime }}\right)}^{\frac{1}{n^{\prime }}}\frac{1}{n^{\prime }}\sigma_{a,e-p}{}^{\frac{1}{n^{\prime }}-1}\right]}^{\frac{1}{5n^{\prime }}}$$

Calculating the maximum stress provided the basis for calculating the strain gradient:

(22) $$\varepsilon_{a,grad}=\left(\frac{\sigma_{a,grad}}{E}\right)+{\left(\frac{\sigma_{a,grad}}{K^{\prime }}\right)}^{\frac{1}{n^{\prime }}}$$

Eventually, after introducing Equation (21) into Equation (22), we obtained:

(23) $$\varepsilon_{a,grad}=\left(\frac{\sigma_{a,e-p}{\left[1+E{\left(\frac{1}{K^{\prime }}\right)}^{\frac{1}{n^{\prime }}}\frac{1}{n^{\prime }}\sigma_{a,e-p}{}^{\frac{1}{n^{\prime }}-1}\right]}^{\frac{1}{5n^{\prime }}}}{E}\right)+{\left(\frac{\sigma_{a,e-p}{\left[1+E{\left(\frac{1}{K^{\prime }}\right)}^{\frac{1}{n^{\prime }}}\frac{1}{n^{\prime }}\sigma_{a,e-p}{}^{\frac{1}{n^{\prime }}-1}\right]}^{\frac{1}{5n^{\prime }}}}{K^{\prime }}\right)}^{\frac{1}{n^{\prime }}}$$

### 2.2. Experimental Tests

The analysis was conducted on 7 materials from different material groups. A part of the research data was obtained from the available literature, and some data were obtained from our own research. The analyzed and tested materials were 10HNAP, based on our research under tension–compression [27] and under bending [28]; 16Mo3, based on data from the literature for tension–compression [29] and on the basis of our own tests for bending [30]; 30CrNiMo3, based on data from the literature for bending [30] and tension–compression [31]; MO58, on the basis of our own tests for bending [32] and for tension–compression [33]; SM45, based on data from the literature for bending [34] and for tension–compression [29]; 2017A-T4, based on our own tests under bending [35] and tension–compression [36]; and 6082-T6, also on the basis of our own tests under bending [37] and tension–compression [38]. Table 1 presents the chemical composition of tested materials, and Table 2 presents the mechanical properties of these materials.

**Figure 4:**
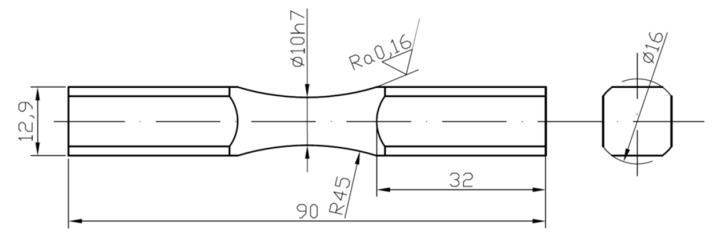
The specimen’s geometry for fatigue testing, in mm.

**Figure 5:**
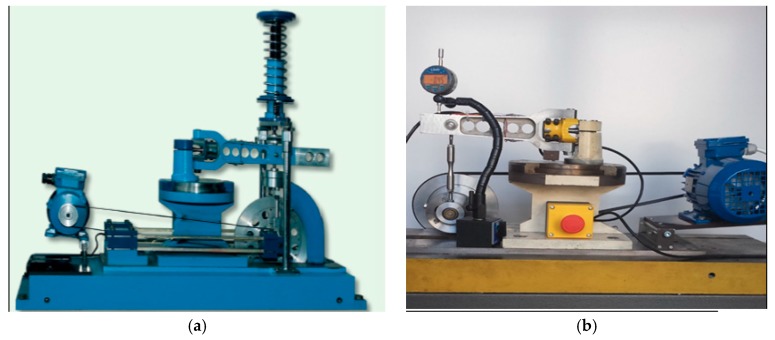
Research stands for fatigue tests with (**a**) controlled moment and (**b**) controlled strain.

Tensile–compression tests were performed under standard conditions on solid round specimens. In fatigue tests, diabolo-type cylindrical specimens with no geometric notch were used, as presented in Figure 4. The tests under cyclic bending conditions at the controlled moment (Figure 5a) were conducted for 10HNAP steel, Mo58 brass, and 2017(A)-T4 and 6082-T6 aluminum alloys. However, cyclic bending tests with controlled strains (Figure 5b) were conducted for 16Mo3 steel and 6082-T6 aluminum.

### 2.3. Analysis

For oscillatory bending, in the first stage, the stress amplitudes from the elastic body model were converted into the elastoplastic body model according to the description and Equations (5)–(7). Then, the calculated stress amplitudes were converted into the model proposed in this paper, which included the stress gradient, according to Equation (21) *σ_a,grad_*, which was the basis for calculating the strain gradient using Equation (23).

We interpreted the results by analyzing the fatigue life scatter, which was used with the help of the logarithm [39]:

(24) $$E=f\left(\log\frac{\varepsilon_{a}}{\varepsilon_{cal}}\right)$$

where $\varepsilon_{cal},$ with the use of the MCB in Equation (4).

The literature [25] suggested determining MSE as:

(25) $$E_{RMS}=\sqrt{\frac{\Sigma {log}^{2}\frac{\varepsilon_{a}}{\varepsilon_{cal}}}{n}}$$

Eventually, to determine the mean scatter, we used:

(26) $$T_{RMS}=10^{E_{RMS}}$$

In the available literature, the subject of scattering has been discussed [14]. We used scattering as one of the methods for comparing models assessing fatigue life.

The scattering was counted in the first stage for tension–compression, then for bending, as described by the strain amplitude according to the elastoplastic model ε_a,e-p_, and then by including strain gradients ε_a,grad_ in relation to the base characteristic determined for tension–compression.

**Figure 6:**
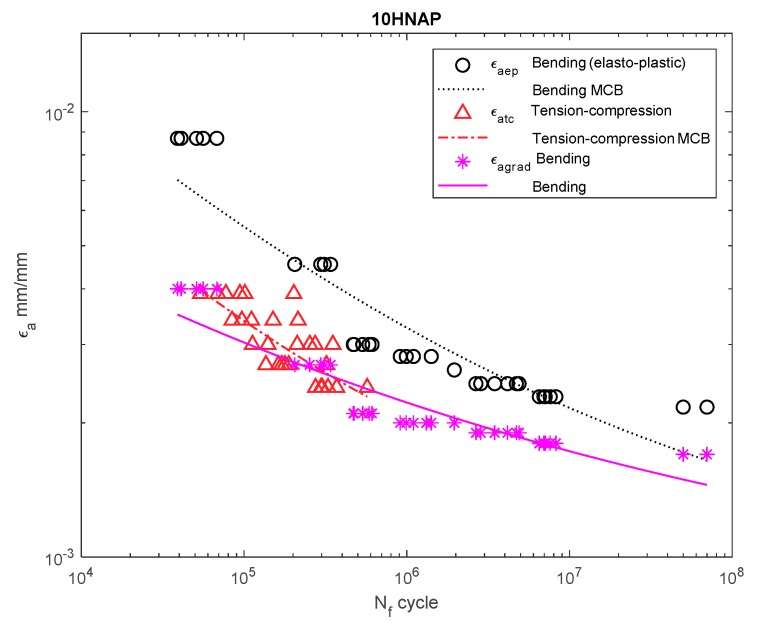
Dependency chart for the strain amplitude $\varepsilon_{a}$ = *f*($N_{f}$) for 10HNAP steel.

**Figure 7:**
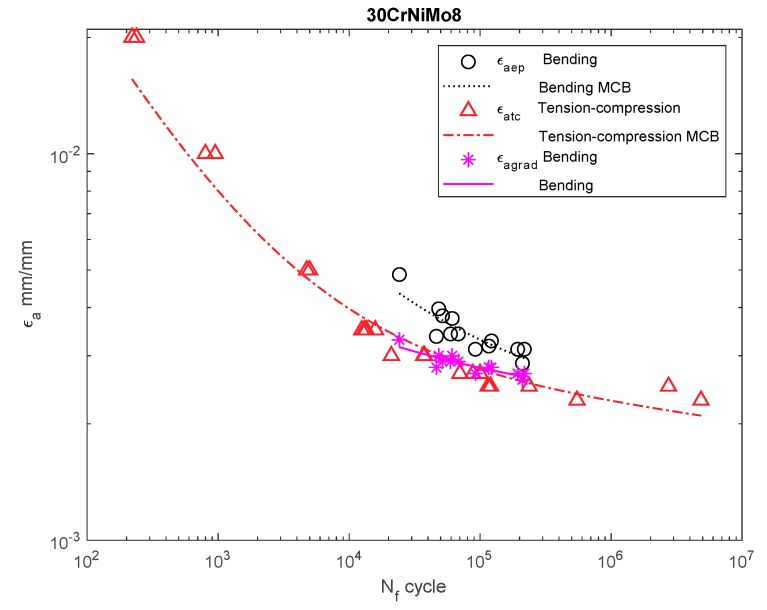
Dependency chart for the strain amplitude *σ_a_* = *f(N_f_)* for 30CrNiMo8 steel.

**Figure 8:**
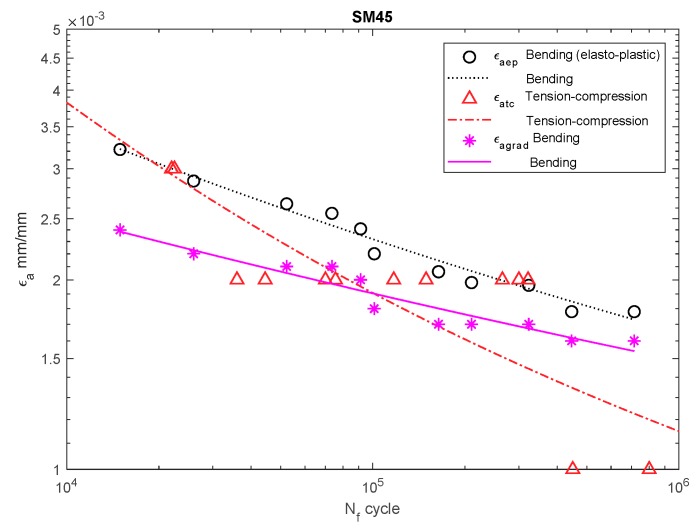
Dependency chart for the strain amplitude *ε_a_* = *f(N_f_)* for SM45 steel.

**Figure 9:**
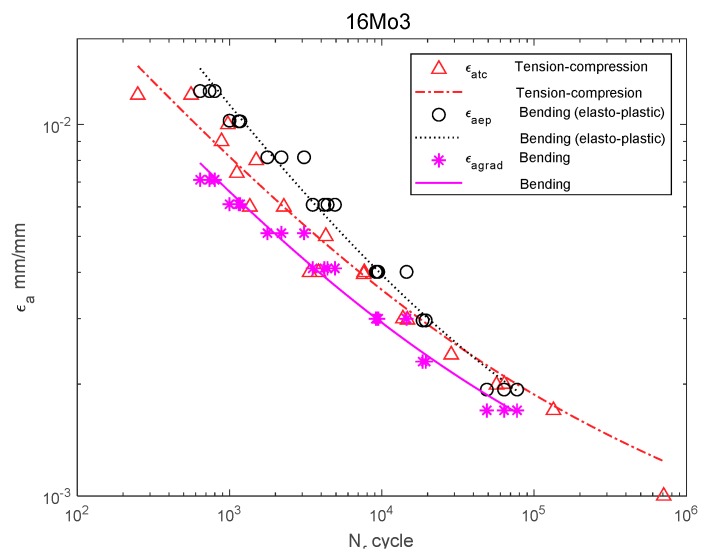
Dependency chart for the strain amplitude $\varepsilon_{a}$ = *f*($N_{f}$) for 16Mo3 steel.

**Figure 10:**
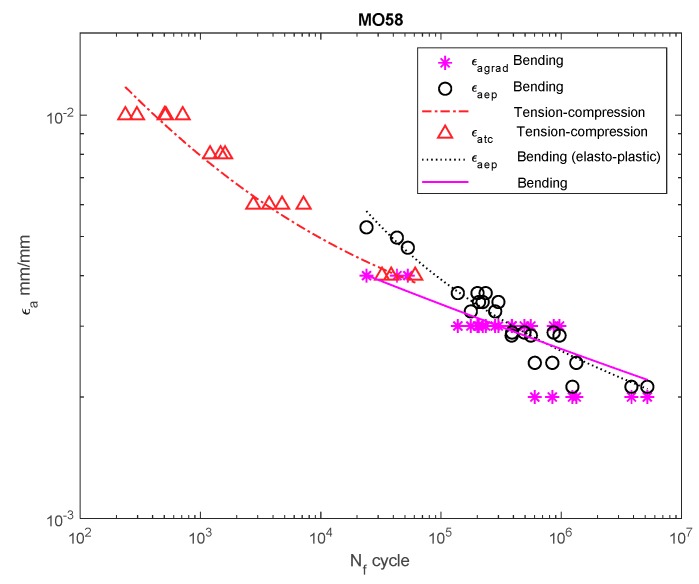
Dependency chart for the strain amplitude $\varepsilon_{a}$ = *f*($N_{f}$) for MO58 steel.

**Figure 11:**
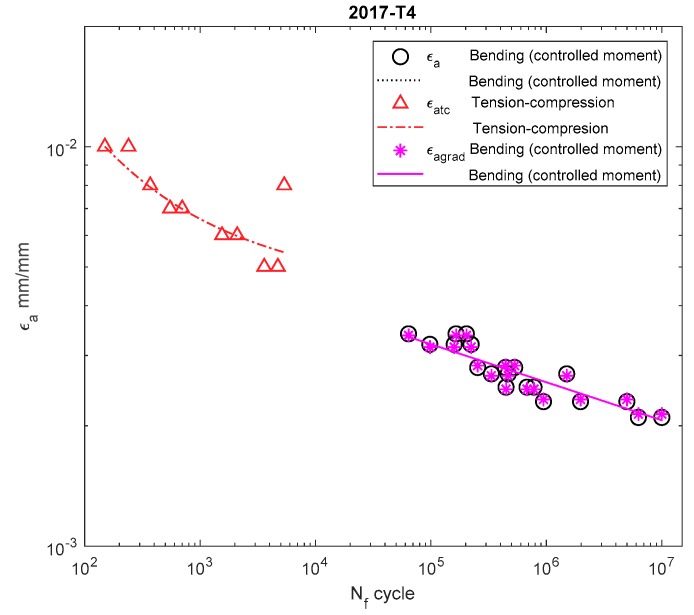
Dependency chart for the strain amplitude $\varepsilon_{a}$ = *f*($N_{f}$) for 2017A-T4 aluminum alloy.

**Figure 12:**
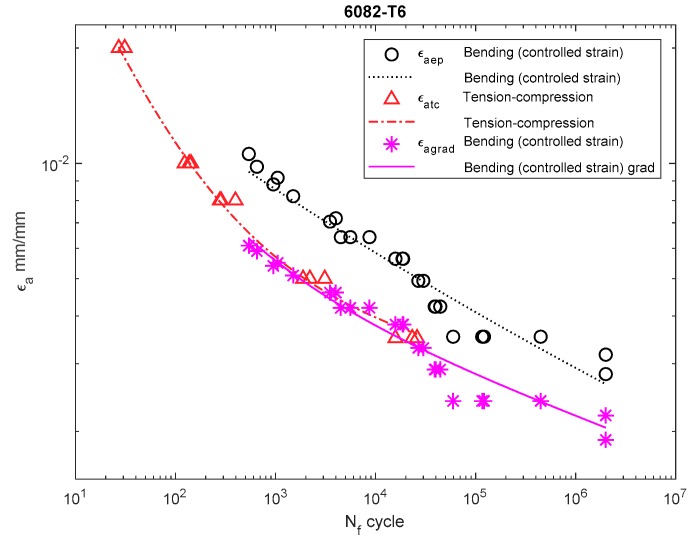
Dependency chart for the strain amplitude $\varepsilon_{a}$ = *f*($N_{f}$) for 6082-T4 steel.

**Figure 13:**
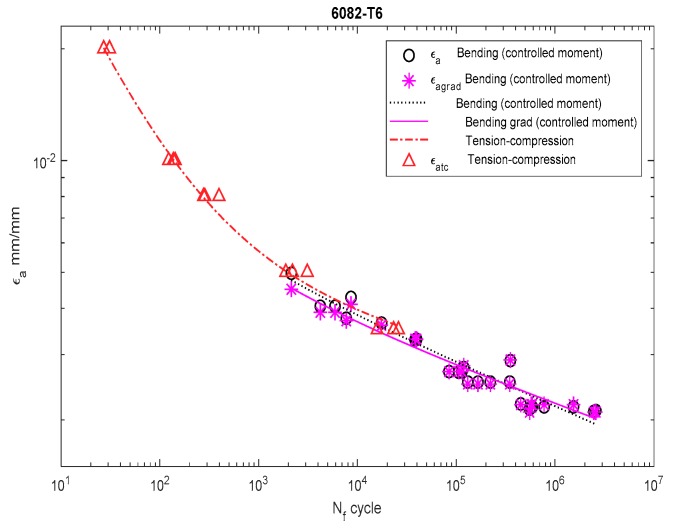
Dependency chart for the strain amplitude $\varepsilon_{a}$ = *f*($N_{f}$) for 6082-T4 steel.

Figure 6, Figure 7, Figure 8, Figure 9, Figure 10, Figure 11, Figure 12 and Figure 13 depict the nominal strain amplitudes, amplitudes determined according to the elastoplastic body model, and, according to the proposed model, the strain gradient against the amplitudes obtained for tension–compression. The inclusion of the elastoplastic model resulted in the reduction of curves, illustrating the results obtained from tests under cyclic bending conditions and the approximation to the curves obtained from tensile–compression testing. On the other hand, considering the gradient effect for most of the analyzed materials led to cyclic bending and to tension–compression.

For 10HNAP steel (Figure 6), the strain amplitude gradient for the results for bending was below the amplitude for the results obtained under bending according to the elastoplastic model. The amplitude of the strain almost coincided with that under tension–compression.

The tests and calculations for 30CrNiMo8 (Figure 7) and SM45C steel (Figure 8) were similar.

For 16Mo3 steel (Figure 9), the strain amplitude was located below the amplitude of the results obtained under tension–compression, and were almost parallel. For MO58 brass (Figure 10), the amplitude according to the new model was also below that of the results obtained under bending according to the elastoplastic model. However, when analyzing MO58 (Figure 10) as well as 2017A-T4 aluminum alloy (Figure 11), comparing the amplitudes obtained under cyclic bending according to all the models with the tensile–compression amplitude was difficult, as the results were in different cycle ranges. The results for 2017-T4 aluminum alloy overlapped each other, both for those obtained under bending according to the elastoplastic and nominal model, as well as according to the new model proposed in Equation (21) presented as $\varepsilon_{agrad}$.

For 6082-T6 aluminum alloy, we analyzed the strain performed for the results obtained under bending at a controlled strain (Figure 12) and at a controlled moment (Figure 13). In both cases, the strain amplitude that included the new solution was below both the results obtained under bending and under tension–compression.

## 3. Discussion

When comparing different models, in order to select the one closest to reality, the fatigue life scatter was analyzed for each of the examined materials. The results are presented in Table 3, Table 4, Table 5, Table 6, Table 7, Table 8 and Table 9. The scatter for 10HNAP steel was the largest for the results obtained under bending. By comparing bending according to the elastoplastic model (*e-p*) and by including the strain gradient (grad), we found that the smallest scatter, and the closest to the tension–compression, were the results produced by the model that included the gradients. When analyzing subsequent materials, we found that this was the same situation for all steel and brass. Thus, for all analyzed steel and brass, satisfactory results were obtained according to the proposed model. For aluminum alloys, no improvement in the scattering was achieved, but the obtained results were acceptable. Scattering for 6082-T4 aluminum alloy also remained in the trend as 2017A-T6 aluminum, but the difference was even smaller, especially between bending under controlled strain and bending with the gradient.

## 4. Conclusions

This paper proposes a model that enables the conversion of strain fatigue characteristics obtained on the basis of a cyclic bending test into equivalents, which coincides with the characteristics obtained in the tensile–compression test. The proposed model is based on the ratio of the stress and strain gradient at a critical location, i.e., on the surface.

From the analyzed materials, we found that the strain amplitudes obtained on the basis of the oscillatory bending test with restraint for a given fatigue life were greater than or equal to those obtained in the tensile–compression test.

For the analyzed materials, we concluded that the strain amplitudes obtained on the basis of the proposed model during the oscillatory bending test with restraint for a given fatigue life were comparable to those obtained from the tensile–compression test, with the exception of 16Mo3 steel.

From the use of scattering, we found that the most reliable calculation results for the oscillatory bending were obtained when including the secant modulus considering plasticity, i.e., the ratio of the stress gradient and the strain gradient.

## Figures and Tables

**Table 1:** Chemical composition of the tested materials (%).

Material	Chemical Composition															
	C	Si	Mn	P	S	Cr	Ni	Mo	Cu	Fe	Mg	Zn	Zr + Ti	Pb	Sn	Al
10HNAP	0.115	0.41	0.71	0.082	0.028	0.81	1.90	0.30	-	The rest	-	-	-	-	-	-
30CrNiMo8	0.3	0.27	0.49	0.019	0.009	3.89	1.90	0.30	-	The rest	-	-	-	-	-	-
SM45C	0.45	0.35	0.64	0.011	0.012	-	-	-	-	The rest	-	-	-	-	-	-
16Mo3	0.19	0.28	0.69	0.019	0.024	-	-	0.33	-	The rest	-	-	-	-	-	-
MO58	-	-	-	-	-	-	Max 0.2	-	56–60	Max 0.5	-	The rest	-	1–3.5	Max 0.5	Max 1
2017(A)	-	0.2–0.8	0.4–1.0	-	-	<0.10	-	-	3.5–4.5	<0.7	0.4–1.0	<0.25	<0.25	-	-	The rest
6082	-	0.7–1.3	0.4–1.0	-	-	<0.25	-	-	<0.1	<0.5	0.6–1.2	<0.2	<0.1	-	-	The rest

**Table 2:** Mechanical properties of the tested and analyzed materials, such as offset yield point (*R_p0.2_*), ultimate tensile strength (*R_m_*), relative elongation (*A*5), and Poisson’s ratio (*ν*).

Material	*R_p_*_0.2_ (MPa)	*R_m_* (MPa)	*A*5 (%)	*ν*
10HNAP	464	566	32	0.29
30CrNiMo8	795	1014	6.3	0.29
SM45C	430	680	15	0.29
16Mo3	335	481	24	0.30
MO58	399	484	-	0.32
2017-T6	395	545	21	0.32
6082-T4	365	385	27.2	0.32

**Table 3:** The cyclic properties of 10HNAP steel.

10HNAP										
Testing Conditions	Material Constant									
	*E* (GPa)	Ramberg–Osgood		Basquin		Manson–Coffin–Basquin (MCB)				T_RMS_
		*K’* (MPa)	*n’*	*A*	*m*	*σ’_f_* (MPa)	*ε’_f_*	*b*	*c*	
Bending	205	-	-	35.96	11.39	-	-	-	-	-
Bending (*e–p*)		-	-	46.64	16.24	675	0.239	−0.052	−0.340	1.616
Tension–compression		853	0.156	29.07	9.57	685	0.245	−0.063	−0.399	1.114
Bending (grad)				58.51	21.41	501	0.0349	−0.039	−2569	1.152

**Table 4:** Cyclic properties of 30CrNiMo3 steel.

30CrNiMo8										
Testing Conditions	Material Constant									
	*E* (GPa)	Ramberg–Osgood		Basquin		MCB				T_RMS_
		*K’* (MPa)	*n’*	*A*	*m*	*σ’_f_* (MPa)	*ε’_f_*	*b*	*c*	
Bending	206	-	-	25.57	7.35	-	-	-	-	
Bending (*e–p*)		-	-	52.07	17.30	911	0.602	−0.045	−0.548	1.229
Tension–compression		972	0.085	49.79	16.64	851	0.471	−0.043	−0.597	1.123
Bending (grad)		-	-	84.25	29.38	693	0.041	−0.027	−0.384	1.159

**Table 5:** Cyclic properties of SM45C.

SM45C										
Testing Conditions	Material Constant									
	*E* (GPa)	Ramberg–Osgood		Basquin		MCB				T_RMS_
		*K’* (MPa)	*n’*	*A*	*m*	*σ’_f_* (MPa)	*ε’_f_*	*b*	*c*	
Bending	201.5	-	-	31.13	10.29	-	-	-	-	
Bending (*e–p*)		-	-	37.78	13.38	671	0.035	−0.071	−0.298	1.246
Tension–compression		1414	0.231	23.69	7.76	1140	0.406	−0.122	−0.53	1.067
Bending (grad)				44.02	16.18	527	0.009	−0.058	−0.224	1.168

**Table 6:** Cyclic properties of 16Mo3 steel.

16Mo3										
Testing Conditions	Material Constant									
	*E* (GPa)	Ramberg–Osgood		Basquin		MCB				T_RMS_
		*K’* (MPa)	*n’*	*A*	*m*	*σ’_f_* (MPa)	*ε’_f_*	*b*	*c*	
Bending	210	-	-	21.07	6.80	-	-	-	-	
Bending (*e–p*)		-	-	24.91	8.40	980	0.769	−0.116	−0.580	1.250
Tension–compression		1038	0.133	27.94	9.67	780	0.233	−0.096	−0.473	1.106
Bending (grad)				26.47	9.05	884	0.071	−0.107	−0.635	1.244

**Table 7:** Cyclic properties of MO58 steel.

MO58										
Testing Conditions	Material Constant									
	*E* (GPa)	Ramberg–Osgood		Basquin		MCB				T_RMS_
		*K’* (MPa)	*n’*	*A*	*m*	*σ’f* (MPa)	*ε’f*	*b*	*c*	
Bending	96.9	-	-	19.98	5.86	-	-	-	-	
Bending (*e–p*)		-	-	25.06	8.04	1175	4.71	−0.110	−0.717	1.169
Tension–compression		723.3	0.121	50.92	18.59	549	0.11	−0.049	−0.434	1.091
Bending (grad)		-	-	25.76	8.3380	936	0.01	−0.095	−0.265	1.155

**Table 8:** Cyclic properties of 2017A-T4 aluminum alloy.

2017A-T4										
Testing Conditions	Material Constant									
	*E* (GPa)	Ramberg–Osgood		Basquin		MCB				T_RMS_
		*K’* (MPa)	*n’*	*A*	*m*	*σ’f* (MPa)	*ε’f*	*b*	*c*	
Bending at the controlled moment	72	-	-	25.59	8.65	738	1	−0.095	0	1.498
Tension–compression		617	0.066	35.55	12.54	553	0.193	−0.044	−0.678	1.158
Bending (grad)		-	-	25.59	8.65	738	1	−0.095	0	1.498

**Table 9:** Cyclic properties of 6082-T6 aluminum alloy.

6082-T6										
Testing Conditions	Material Constant									
	*E* (MPa)	Ramberg–Osgood		Basquin		MCB				T_RMS_
		*K*’ (MPa)	*n’*	A	*m*	*σ’_f_* (MPa)	*ε’_f_*	*b*	*c*	
Bending at the controlled moment	76.998	-	-	23.7053	7.9930	905	0.0530	−0.116	−0.610	1.179
Bending at the controlled moment (grad)		-	-	-	-	687	0.0419	−0.096	−0.516	1.183
Bending at the controlled strain		-	-	25.1731	8.6950	768	0.2836	−0.105	0.649	1.150
Bending at the controlled strain (grad)		-	-	26.47	9.28	696	0.0835	−0.098	−0.548	1.153
Tension–compression		616	0.099	37.5945	13.7902	533	0.185	−0.065	−0.634	1.050
